# High-Capacity Reversible Data Hiding in Encrypted Images with Flexible Restoration

**DOI:** 10.3390/jimaging8070176

**Published:** 2022-06-21

**Authors:** Eichi Arai, Shoko Imaizumi

**Affiliations:** 1 Graduate School of Science and Engineering, Chiba University, 1-33 Yayoicho, Chiba 263-8522, Japan; eichin-5240@chiba-u.jp; 2 Graduate School of Engineering, Chiba University, 1-33 Yayoicho, Chiba 263-8522, Japan

**Keywords:** reversible data hiding in encrypted images, high hiding capacity, flexible restoration, bit-plane partition, image encryption

## Abstract

In this paper, we propose a novel reversible data hiding in encrypted images (RDH-EI) method that achieves the highest hiding capacity in the RDH-EI research field and full flexibility in the processing order without restrictions. In the previous work in this field, there exist two representative methods; one provides flexible processing with a high hiding capacity of 2.17 bpp, and the other achieves the highest hiding capacity of 2.46 bpp by using the BOWS-2 dataset. The latter method has critical restrictions on the processing order. We focus on the advantage of the former method and introduce two efficient algorithms for maximizing the hiding capacity. With these algorithms, the proposed method can predict each pixel value with higher accuracy and refine the embedding algorithm. Consequently, the hiding capacity is effectively enhanced to 2.50 bpp using the BOWS-2 dataset, and a series of processes can be freely conducted without considering any restrictions on the order between data hiding and encryption. In the same way, there are no restrictions on the processing order in the restoration process. Thus, the proposed method provides flexibility in the privileges requested by users. Experimental results show the effectiveness of the proposed method in terms of hiding capacity and marked-image quality.

## 1. Introduction

In recent years, with the development of social networking services and cloud services, images are increasingly being uploaded to external servers or services for share and disclosure. However, this leads to leakage of personal information and copyright infringement due to unauthorized secondary use. In response to this situation, reversible data hiding (RDH), which is an image protection technique, has attracted attention. RDH allows an image owner to embed arbitrary data (hereafter a payload), e.g., copyright and authentication information, into an image without increasing the original file size. RDH methods can completely retrieve original images by extracting the payload from marked images [1,2,3,4,5]. This feature is practically effective not only for natural images but also for medical, military, and satellite images. RDH methods have been commonly applied to plane images, but, recently, RDH in encrypted images (RDH-EI) methods have also been actively studied [6,7,8,9,10,11,12,13,14,15,16]. With RDH-EI methods, we assume that an image owner first encrypts a target image and then sends it to a third party such as a service provider. The third party then embeds a payload, such as server information, access history, and annotation data. Therefore, a high hiding capacity is one of the requirements in the RDH-EI research field. Some RDH-EI methods have also been proposed for medical images with DICOM format and HDR images [17,18].

In an RDH-EI method proposed by Ma et al. [7], an image owner first divides an original image into two areas; one is used for data hiding, and the other is used for data storage for reversibility. The least significant bits (LSBs) of the former area are reversibly embedded into the latter area, so these LSBs are used for data hiding. The image owner can also use multiple bit-planes for data hiding depending on the area ratio. Then, the entire image is encrypted and sent to a third party. The third party embeds a payload into the LSB plane by bit substitution and derives a marked encrypted image. This method can obtain a high-quality marked image, which still contains a payload, through decryption without data extraction. However, the hiding capacity is at the most 0.5 bpp. The hiding capacity of any other methods using LSBs as data hiding area is limited to less than 1 bpp [8,9]. Another RDH-EI method proposed by Xu et al. [10] independently conducts data hiding and encryption processes without dividing an image into two areas, but the hiding capacity of this method is still less than 1 bpp. Wu et al. enhanced the hiding capacity of the Ma et al.’s method [7] by introducing the adaptive bit-plane partition [11]. Specifically, an original image is divided into two areas; one contains lower bit-planes, i.e., less significant bit-planes, and the other contains upper bit-planes, i.e., more significant bit-planes. The original bit values of the lower bit-planes are embedded into the upper bit-planes, and then the upper bit-planes are encrypted. The lower bit-planes are used for data hiding. This method achieves a high hiding capacity of more than 2 bpp. Additionally, since encryption for upper bit-planes and data hiding for lower bit-planes are conducted independently, decryption and data extraction can also be performed without restriction on their order. Hereafter, we call this method the RDH-BPP method. On another front, several unique approaches have been developed to compress a marked encrypted image [15,16]. These methods use one type of encryption and then compression systems [19,20,21] that can efficiently compress an encrypted image using an image coding standard.

Puteaux et al. proposed a high capacity RDH-EI method that introduces prediction and replacement using most significant bits (MSBs) instead of LSBs [12]. However, this method cannot fully retrieve the original image in many cases, so Hirasawa et al. extended Puteaux et al.’s method [12] and attained full reversibility by defining precise conditions for MSB substitution [13]. Later, Puteaux et al. proposed a more efficient RDH-EI method that guarantees full reversibility and considerably enhances the hiding capacity to 2.4 bpp on average [14] by using the BOWS-2 dataset. To the best of our knowledge, this capacity is the highest one in the RDH-EI research field. The recursive process using MSB prediction contributes such a high capacity. This method ensures full reversibility through a pixel-value modification process based on an error detection algorithm. Hereafter, we call this method the RDH-MSB method. The RDH-MSB method, however, has restrictions on the processing order, and the order cannot be changed. In the restoration process, for instance, the order needs to be data extraction and then decryption, so we cannot decrypt a marked encrypted image without data extraction. This limits the range of practical applications.

In this paper, we propose a novel RDH-EI method that achieves both the highest hiding capacity and processing flexibility. We focus on the flexible processing sequence in the RDH-BPP method and develop prediction and embedding algorithms to enhance the hiding capacity. The proposed method, as with the RDH-BPP method, divides an original image into two areas by bit-plane partition, and thus the encryption and data hiding processes are totally independent from each other. Accordingly, there are no restrictions on the processing order, and the method can be applied to a wide range of applications. Through our experiments, we confirmed the effectiveness of the method in terms of hiding capacity and marked-image quality.

## 2. Related Works

As mentioned earlier, the proposed method uses an effective feature of the RDH-BPP method [11] and achieves a higher hiding capacity than the RDH-MSB method [14]. We explain the RDH-BPP and RDH-MSB methods as follows.

### 2.1. Bit-Plane-Partition-Based RDH-EI Method

The RDH-BPP method [11] is an RDH-EI method using bit-plane partition. An original image is divided into two areas on the basis of bit-planes: $I_{1}$ containing upper bit-planes and $I_{2}$ containing lower bit-planes. The encryption process is conducted on $I_{1}$, while the data hiding process is conducted on $I_{2}$. Such independent processes contribute to making the processing order flexible. In the restoration process, specifically, the decryption process can be conducted without data extraction, even when the encryption process has been performed before data hiding. In this case, we can obtain high quality images containing a payload.

**Figure 1:**
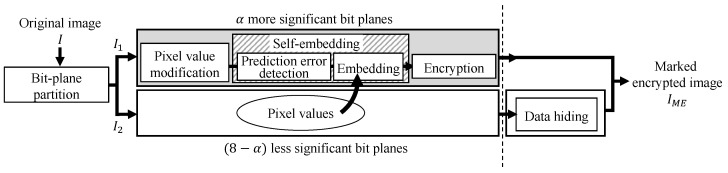
Block diagram of proposed method and RDH-BPP method [11].

Figure 1 shows an outline of this method, where $I_{1}$ contains α bits of upper bit-planes, and $I_{2}$ contains 8−α bits of lower bit-planes. $I_{2}$ is used as a data hiding area, so pixel values need to be stored into $I_{1}$. We call this process the self-embedding process. The self-embedding algorithm is analogous to a part of another previous RDH method [2]. We calculate prediction values for each pixel in $I_{1}$, and the pixel values of $I_{2}$ are embedded into $I_{1}$ on the basis of another embedding algorithm, for which a fundamental prediction error expansion with histogram shifting (PEE-HS) method [3] has been extended. The detailed steps of the self-embedding process are described below. Note that the image size is M×N.

**Figure 2:**
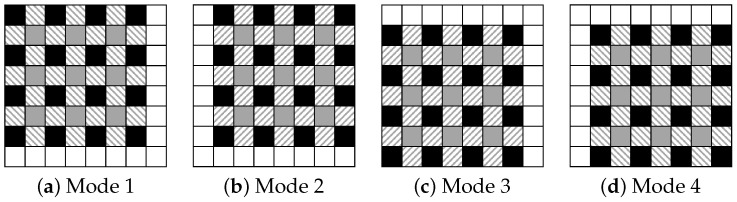
Four prediction modes.

**Figure 3:**
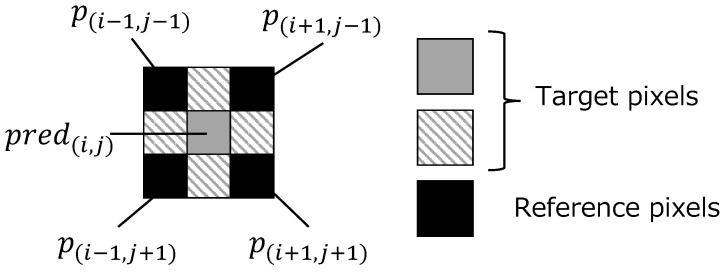
Target and reference pixels in $I_{1}$.

**Step1-1:**  Prepare four modes for prediction as shown in Figure 2, and define the processing order of these modes.
**Step1-2:**  Derive prediction values for the target pixels in $I_{1}$ using the reference pixels as shown in Figure 3. For the target pixels $p_{\left(i,j-1\right)}$ and $p_{\left(i-1,j\right)}$ (1≤i<M, 1≤j<N), the prediction values $pred_{\left(i,j-1\right)}$ and $pred_{\left(i-1,j\right)}$ are obtained by
  (1) $$pred_{\left(i,j-1\right)}=\frac{p_{\left(i-1,j-1\right)}+p_{\left(i+1,j-1\right)}}{2},$$
  (2) $$pred_{\left(i-1,j\right)}=\frac{p_{\left(i-1,j-1\right)}+p_{\left(i-1,j+1\right)}}{2}.$$
  In contrast, to derive the prediction value $pred_{\left(i,j\right)}$, first calculate the interpolated values $g_{1}$ and $g_{2}$ using pairs of two diagonal reference pixels:
  (3) $$\left\{\begin{array}{c} g_{1}=\frac{p_{\left(i-1,j-1\right)}+p_{\left(i+1,j+1\right)}}{2},\\ g_{2}=\frac{p_{\left(i+1,j-1\right)}+p_{\left(i-1,j+1\right)}}{2}. \end{array}\right.$$
  Using $g_{1}$, $g_{2}$ and the mean value μ of the four reference pixels, the variances $\delta_{1}$ and $\delta_{2}$ between two diagonal reference pixels are derived by
  (4) $$\left\{\begin{array}{c} \delta_{1}=\frac{{\left(p_{\left(i-1,j-1\right)}-\mu \right)}^{2}+{\left(g_{1}-\mu \right)}^{2}+{\left(p_{\left(i+1,j+1\right)}-\mu \right)}^{2}}{3},\\ \delta_{2}=\frac{{\left(p_{\left(i+1,j-1\right)}-\mu \right)}^{2}+{\left(g_{2}-\mu \right)}^{2}+{\left(p_{\left(i-1,j+1\right)}-\mu \right)}^{2}}{3}. \end{array}\right.$$
  Finally, the prediction value $pred_{\left(i,j\right)}$ is given by
  (5) $$pred_{\left(i,j\right)}=g_{1}\cdot \frac{\delta_{2}}{\delta_{1}+\delta_{2}}+g_{2}\cdot \frac{\delta_{1}}{\delta_{1}+\delta_{2}}.$$
**Step1-3:**  Derive the prediction errors $e_{\left(i,j\right)}$ as follows:
  (6) $$e_{\left(i,j\right)}=pred_{\left(i,j\right)}-p_{\left(i,j\right)}.$$
**Step1-4:**  Using the extended PEE-HS method [2], the original bit values in $I_{2}$ and additional information for reversibility are embedded into $I_{1}$. Note that the embedding algorithm in [2] has been extended from the original PEE-HS method [3] in terms of the embedding efficiency.
**Step1-5:**  Repeat Steps 1–2 to 1–4 for the four modes until all the bits in $I_{2}$ are embedded.
**Step1-6:**  If a part of the bits in $I_{2}$ have not been embedded into $I_{1}$, repeat Steps 1–2 to 1–5.
**Step1-7:**  Replace the pixel values of $I_{2}$, where the original bit values have been embedded into $I_{1}$, with 0.

The self-embedding process guarantees reversibility. If the total amount of original bits in $I_{2}$ is larger than the hiding capacity of $I_{1}$, a part of $I_{2}$ cannot serve as the data hiding area. In this case, the bits of $I_{2}$ that cannot be embedded into $I_{1}$ or are located in the same row with the first bit to be unembedded are not replaced with 0 and are omitted from the data hiding area. The row and bit-plane numbers of the last embedded bit are stored as additional information.

After the self-embedding process, we encrypt $I_{1}$ using a pixel-by-pixel encryption algorithm, and an encrypted image is derived. The encrypted image is sent to a third party such as a service provider. The third party embeds an arbitrary payload into $I_{2}$ of the encrypted image using bit substitution. Here, the embeddable bits of $I_{2}$ are defined by the self-embedding process and have a value of 0. The payload cannot be embedded into bits with a value of 1.

### 2.2. MSB-Prediction-Based RDH-EI Method

The RDH-MSB method [14] has a high hiding capacity and guarantees perfect reversibility. In this method, each bit-plane is processed recursively, in an order from MSB to LSB. Consequently, the hiding capacity is 2.46 bpp on average by using the BOWS-2 dataset, which is the highest among any related work. This method guarantees reversibility by using a pixel value modification process based on prediction errors.

**Figure 4:**
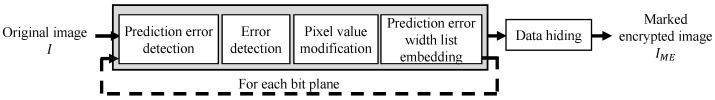
Block diagram of an RDH-MSB method [14].

An outline of the RDH-MSB method is shown in Figure 4. An original image *I* is composed of eight bit-planes $B^{\left[1,8\right]}$, and $B^{\left[1\right]}$ represents the MSB plane. The *k*-th bit-plane $B^{\left[k\right]}$, where *k* ranges from 1 to 8, is a target plane to be processed, and the lower bit-planes $B^{\left[k+1,8\right]}$ are used for the reversibility of $B^{\left[k\right]}$.

First, an image owner encrypts target images with the following procedure.

**Enc1:**  Assign k=1.
**Enc2:**  Derive prediction values $pred_{\left(i,j\right)}^{\left[k,8\right]}$ for target partial pixels $p_{\left(i,j\right)}^{\left[k,8\right]}$, simply called pixels hereafter, using the median edge detection (MED) method:
  (7) $$\;pred_{\left(i,j\right)}^{\left[k,8\right]}=\left\{\begin{array}{cc} min(p_{\left(i-1,j\right)}^{\left[k,8\right]},p_{\left(i,j-1\right)}^{\left[k,8\right]}) & \\ \;\;if\;p_{\left(i-1,j-1\right)}^{\left[k,8\right]}\geq max\left(p_{\left(i-1,j\right)}^{\left[k,8\right]},p_{\left(i,j-1\right)}^{\left[k,8\right]}\right),\\ max(p_{\left(i-1,j\right)}^{\left[k,8\right]},p_{\left(i,j-1\right)}^{\left[k,8\right]}) & \\ \;\;if\;p_{\left(i-1,j-1\right)}^{\left[k,8\right]}\leq min\left(p_{\left(i-1,j\right)}^{\left[k,8\right]},p_{\left(i,j-1\right)}^{\left[k,8\right]}\right),\\ p_{\left(i-1,j\right)}^{\left[k,8\right]}+p_{\left(i,j-1\right)}^{\left[k,8\right]}-p_{\left(i-1,j-1\right)}^{\left[k,8\right]} & \;otherwise. \end{array}\right.$$
  Here, $pred_{\left(0,j\right)}^{\left[k,8\right]}$ and $pred_{\left(i,0\right)}^{\left[k,8\right]}$ cannot be obtained by Equation (7), so define different equations for them:
  (8) $$pred_{\left(0,j\right)}^{\left[k,8\right]}=p_{\left(0,j-1\right)}^{\left[k,8\right]},$$
  (9) $$pred_{\left(i,0\right)}^{\left[k,8\right]}=p_{\left(i-1,0\right)}^{\left[k,8\right]}.$$
**Enc3:**  Calculate prediction errors $e_{\left(i,j\right)}^{\left[k,8\right]}$ for each pixel and detect errors. Note that the errors prevent the algorithm from ensuring reversibility.
**Enc4:**  For each pixel, where an error has been detected, modify the pixel value so that the prediction error is translated into $2^{7-k}$. Here, define the difference between the original and modified pixel values as the prediction-error width.
**Enc5:**  Compare the size of a series of the prediction-error widths $W^{k}$ and the hiding capacity of $B^{\left[k\right]}$. If $W^{k}$ is smaller than the hiding capacity, the current bit-plane $B^{\left[k\right]}$ is embeddable. When k≥2, replace the flag bit $p_{\left(1,0\right)}^{\left[k-1\right]}$ with 1. Here, the flag bit $p_{\left(1,0\right)}^{\left[k\right]}$ denotes whether the next bit-plane will be marked or unmarked. Otherwise, $B^{\left[k\right]}$ is unembeddable, so restore the original pixel values, which are modified in Enc 4, and encrypt all bit-planes $B^{\left[k,8\right]}$.
**Enc6:**  Generate pseudo-random number sequences, and encrypt both of the bit values $p_{\left(i,j\right)}^{\left[k\right]}$ and $W^{k}$ using an exclusive-or operation. The encrypted ones ${\hat{p}}_{\left(i,j\right)}^{\left[k\right]}$ and ${\hat{W}}^{k}$ can be obtained.
**Enc7:**  Following a top-left bit $p_{\left(0,0\right)}^{\left[k\right]}$ and flag bit $p_{\left(1,0\right)}^{\left[k\right]}$, embed ${\hat{W}}^{k}$ and an end flag into $B^{\left[k\right]}$ by bit substitution.
**Enc8:**  Repeat the steps from Enc 2 to 7 after incrementing *k* by one (1≤k≤7).

On the other hand, the third party can embed an arbitrary payload depending on the hiding capacity.

**Figure 5:**
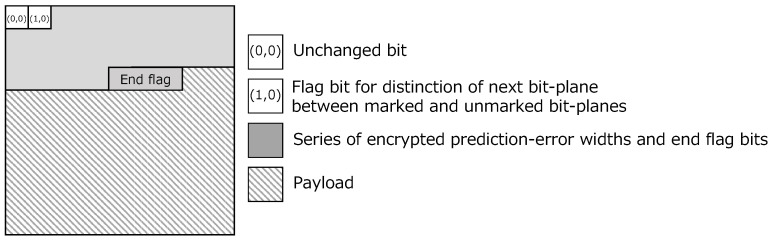
Structure of a single bit-plane in a marked encrypted image.

**Hid1:**  Obtain the hiding capacity from the flag bit $p_{\left(1,0\right)}^{\left[k\right]}$ and end flags.
**Hid2:**  Embed the payload into the embeddable area in each bit-plane by bit substitution, and derive a marked encrypted image, where a single bit-plane is shown in Figure 5.

This method guarantees perfect reversibility through error detection and achieves a high hiding capacity through recursive processing for multiple bit-planes. However, since the encryption and data hiding processes are not independent from each other, the decryption process has to always be conducted after data extraction.

## 3. Proposed Method

We propose a novel RDH-EI method that has the advantages of both the RDH-BPP and RDH-MSB methods [11,14]. The proposed method has unique algorithms for the self-embedding process. We focus on the MED method for prediction and improve the PEE-HS method [3] for data hiding. We describe the detailed procedures.

### 3.1. Encryption and Data Hiding Process

The proposed method uses the MED method to calculate prediction values in the self-embedding process. The MED method has a higher prediction accuracy than the prediction algorithm used in the RDH-BPP method [11]. Furthermore, the proposed method enhances the hiding capacity of $I_{1}$ by refining the self-embedding algorithm that stores the original bits of $I_{2}$ into $I_{1}$. The outline of the proposed method is analogous to that of the RDH-BPP method [11] as shown in Figure 1. We explain the detailed steps of the self-embedding process as follows.

**Step3-1:**  Split an original image into two areas $I_{1}$ and $I_{2}$ by bit-plane partition. $I_{1}$ contains α bits of upper bit-planes and is used for encryption, while $I_{2}$ contains 8−α bits of lower bit-planes and is used for data hiding.
**Step3-2:**  Derive the prediction value $pred_{\left(i,j\right)}$ for each pixel $p_{\left(i,j\right)}$ in $I_{1}$ using the MED method:
  (10) $$\;pred_{\left(i,j\right)}=\left\{\begin{array}{cc} min(p_{\left(i-1,j\right)},p_{\left(i,j-1\right)}) & \\ \;\;if\;p_{\left(i-1,j-1\right)}\geq max\left(p_{\left(i-1,j\right)},p_{\left(i,j-1\right)}\right),\\ max(p_{\left(i-1,j\right)},p_{\left(i,j-1\right)}) & \\ \;\;if\;p_{\left(i-1,j-1\right)}\leq min\left(p_{\left(i-1,j\right)},p_{\left(i,j-1\right)}\right),\\ p_{\left(i-1,j\right)}+p_{\left(i,j-1\right)}-p_{\left(i-1,j-1\right)} & \;otherwise. \end{array}\right.$$
**Step3-3:**  Derive the prediction error $e_{\left(i,j\right)}$ by Equation (6).
**Step3-4:**  Embed a part of the bit values *b* of $I_{2}$ into pixels in $I_{1}$, where $e_{\left(i,j\right)}=0$:
  (11) $$e_{\left(i,j\right)}^{\prime }=\left\{\begin{array}{cc} e_{\left(i,j\right)} & if\;e_{\left(i,j\right)}<0,\\ e_{\left(i,j\right)}+b & if\;e_{\left(i,j\right)}=0,\\ e_{\left(i,j\right)}+1 & if\;e_{\left(i,j\right)}>0, \end{array}\right.$$
  where $e_{\left(i,j\right)}^{\prime }$ denotes the prediction error after the embedding process in this step.
**Step3-5:**  Explore two bins $max_{n}$ and $max_{p}$ ($max_{n}\leq 0$ and $max_{p}>0$) with the highest frequency from a prediction-error histogram.
**Step3-6:**  Embed the remaining bit values *b* of $I_{2}$ into pixels in $I_{1}$, where $e_{\left(i,j\right)}^{\prime }=max_{n}$ or $max_{p}$:
  (12) $$e_{\left(i,j\right)}^{\prime \prime }=\left\{\begin{array}{cc} e_{\left(i,j\right)}^{\prime }-1 & if\;e_{\left(i,j\right)}^{\prime }<max_{n},\\ e_{\left(i,j\right)}^{\prime }-b & if\;e_{\left(i,j\right)}^{\prime }=max_{n},\\ e_{\left(i,j\right)}^{\prime } & if\;max_{n}<e_{\left(i,j\right)}^{\prime }<max_{p},\\ e_{\left(i,j\right)}^{\prime }+b & if\;e_{\left(i,j\right)}^{\prime }=max_{p},\\ e_{\left(i,j\right)}^{\prime }+1 & if\;e_{\left(i,j\right)}^{\prime }>max_{p}, \end{array}\right.$$
  where $e_{\left(i,j\right)}^{\prime \prime }$ denotes the prediction error after the embedding process in this step.
**Step3-7:**  Repeat Steps 3–5 and 3–6 until all of *b* are embedded, and then replace *b* with 0.

If the total amount of the original bits in $I_{2}$ is larger than the hiding capacity of $I_{1}$, a part of $I_{2}$ is excluded from the embeddable area. In this case, the same process as the RDH-BPP method [11] must be conducted for reversibility.

### 3.2. Decryption and Data Extraction Process

**Figure 6:**
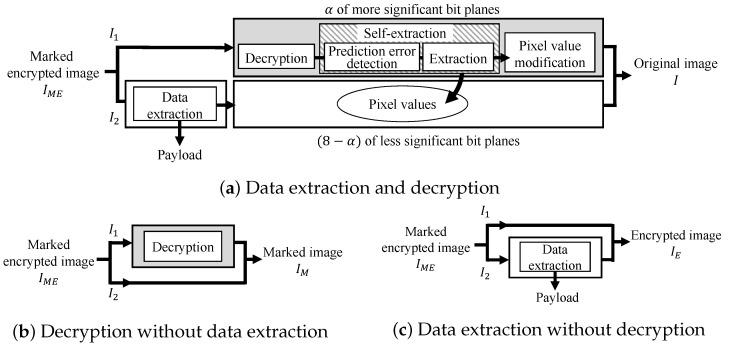
Restoration process of the proposed method.

Here, we explain the decryption and data extraction processes. In the proposed method, encryption for $I_{1}$ and data hiding for $I_{2}$ are conducted independently, so we can conduct decryption and data extraction without any restriction on their order. Consequently, there exist three patterns for the restoration process as shown in Figure 6. In all patterns, a marked encrypted image $I_{ME}$ is first divided into $I_{1}$ and $I_{2}$.

Figure 6a shows a pattern for retrieving the original image and payload. In this case, first, the payload is extracted from $I_{2}$, and the decryption process is applied to $I_{1}$. Here, these processes can be conducted in parallel. Subsequently, the original bits in $I_{2}$ are retrieved from $I_{1}$ by a self-extraction process, which is the reverse of the self-embedding process. Then, the pixel values of $I_{1}$, which have been adjusted for reversibility, are replaced with the original ones. Finally, the original image *I* can be recovered by integrating $I_{1}$ and $I_{2}$.

Figure 6b shows another pattern, where a user has the privilege to decrypt $I_{ME}$ but cannot extract the payload. In this case, the user can directly decrypt $I_{ME}$ without data extraction and obtain a marked image $I_{M}$ still containing the payload.

In the third case, a user is allowed to extract the payload but cannot access the image content as shown in Figure 6c. The user can obtain the payload from $I_{ME}$ and an encrypted image $I_{E}$.

## 4. Experimental Results

**Figure 7:**
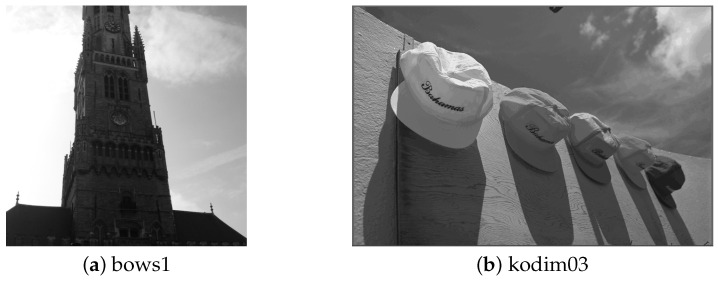
Test images.

**Figure 8:**
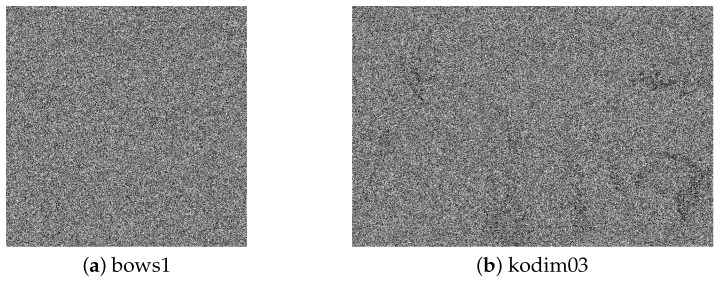
Marked encrypted images.

We confirmed the performance of the proposed method in terms of hiding capacity and marked-image quality. In the experiments, we used two datasets: BOWS-2 [22] and Kodak Lossless True Color Image Suite [23]. The former dataset consists of 10,000 grayscale images with 512×512 pixels, while the latter dataset consists of 24 color images with 512×768 or 768×512 pixels. For the latter dataset, we converted RGB to grayscale. Figure 7 shows examples of the test images. Using the proposed and RDH-BPP methods, the original images were divided into two areas using bit-plane partition: α bits of upper bit-planes and 8−α bits of lower bit-planes. α controls the hiding capacity for payloads and is defined as α∈{7,6,5}. In the RDH-BPP method, there are 24 combinations of orders in which the four modes can be used for prediction. In our simulation, all test images were processed in the fixed order of a, b, c, and d shown in Figure 2. Note that we had confirmed that the processing sequence of the modes has little effect on the results. Figure 8 shows the marked encrypted images obtained by the proposed method.

### 4.1. Hiding Capacity

**Figure 9:**
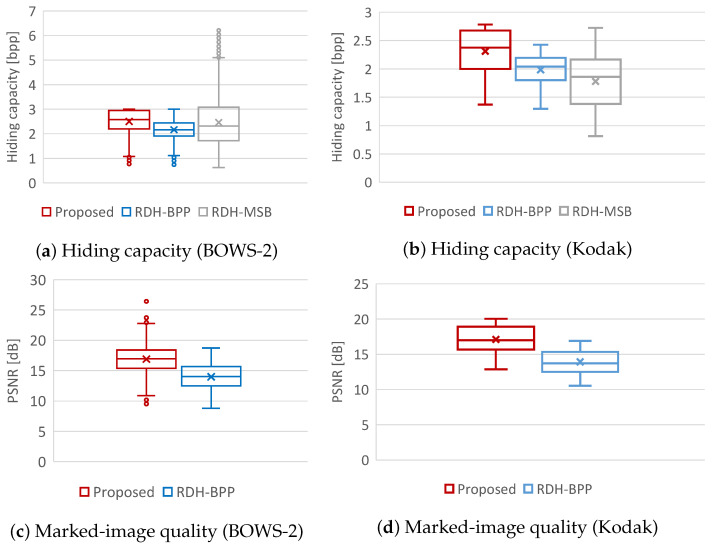
Evaluation results through experiments.

We first compared the hiding capacity among the proposed, RDH-BPP [11], and RDH-MSB [14] methods. Figure 9a,b illustrate the hiding capacity for the all test images of each dataset, and Table 1 shows the average hiding capacity for each method. The proposed method obviously outperformed the RDH-BPP method in both datasets. As can be seen in Figure 9a, the RDH-MSB method has achieved an enormously high hiding capacity for some images in the BOWS-2 dataset. However, images that can handle such a high hiding capacity are low in number. Furthermore, when using the Kodak dataset, the RDH-MSB method has the lowest capacity in these three methods as shown in Figure 9b. The RDH-MSB method attains the highest hiding capacity in several images, but the hiding capacity of this method strongly depends on image features and has a large variance. Comparing the average hiding capacity in Table 1, the proposed method has the highest hiding capacity in both datasets and provides stable high-performance.

Note that the proposed method has processing flexibility as mentioned in Section 3.2, while the RDH-MSB method [14] has a strict restriction on the processing order and prohibits decryption without data extraction. Through this experiment, it has been demonstrated that the proposed method is one of the best RDH-EI methods in terms of hiding capacity.

### 4.2. Marked-Image Quality

**Figure 10:**
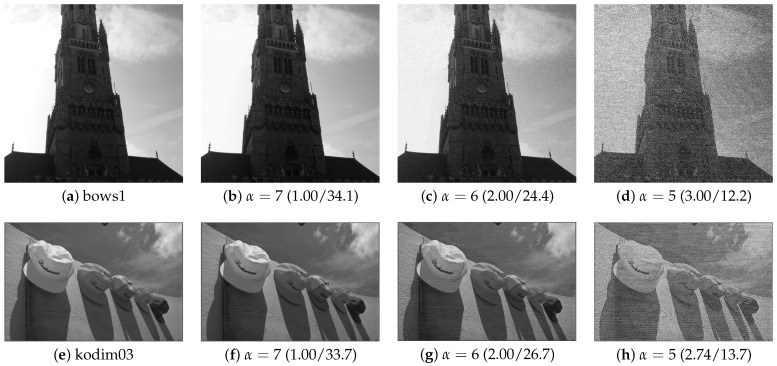
Marked images obtained by the proposed method (hiding capacity [bpp]/PSNR [dB]).

We then evaluated the marked-image quality using PSNR and SSIM. Figure 10 shows the marked images obtained by the proposed method under different α values. Using Figure 9c,d and Table 2, we compare the marked-image quality between the proposed and RDH-BPP methods. In this experiment, we controlled the payload amount for each image so that the proposed method had a comparable amount of payload with the RDH-BPP method. The average payload amounts were 2.17 bpp in the BOWS-2 dataset and 1.99 bpp in the Kodak dataset, respectively. Note that the RDH-MSB method [14] cannot perform decryption without data extraction, so marked images are not derived. It is evident that the proposed method enhanced the marked-image quality in terms of both PSNR and SSIM compared with the RDH-BPP method.

Relative contrast error (RCE) was applied in order to consider the variation in brightness contrast [24]:

(13) $$RCE=0.5+\frac{{std}_{V^{\prime }}-std_{V}}{255},$$

where $std_{V}$ and $std_{V^{\prime }}$ denote the standard deviations in brightness for the original and marked images, respectively. The RCE values ranges from 0 to 1, where 0.5 represents a reference value. Contrast distortion is prominently visible when the absolute difference between the computed RCE and reference values is large. As can be seen in Table 2, the proposed method alleviated the image distortion equally in terms of the contrast. In comparison, the contrast distortion with the RDH-BPP method was caused by the iterative process. A detailed discussion about the iterative process will be given in Section 4.3.

### 4.3. Discussion

We first discuss the advantages of our method in a comparison with the related work [11,14]. In Table 3, we summarize the features of the proposed, RDH-BPP, and RDH-MSB methods. The proposed method achieves all three features, while the related work sacrifices at least one of the features to satisfy the others.

Firstly, we focus on the hiding capacity. The proposed method is based on the RDH-BPP method, while the self-embedding process is widely different from this method. In our method, the MED algorithm is adopted for prediction, and the fundamental PEE-HS method [3] is extended for self-embedding. With the extension, the proposed method enhances the hiding capacity of the RDH-BPP method in both of the two datasets. In the BOWS-2 dataset, the hiding capacity of our method was comparable to that of the RDH-MSB method, which is highest in the RDH-EI research field to the best of our knowledge. In the Kodak dataset, however, the hiding capacity of the RDH-MSB method was lowest in the three methods. Consequently, the proposed method has the best performance among RDH-EI methods on the hiding capacity front.

Next, we consider the flexibility of the processing order. The proposed method has been extended from the RDH-BPP method without losing the advantages; thus, it has the flexibility without any restriction on the processing order. The encryption and data hiding processes are independent from each other, so the encryption/data hiding and decryption/data extraction orders are completely arbitrary. This feature makes it possible to expand the range of practical applications. Additionally, the lack of restrictions on the restoration process provides the following advantages in addition to retrieving the original image *I*. A user with the privilege to only decrypt marked encrypted images can decrypt a marked encrypted image $I_{ME}$ without data extraction and obtain the marked image $I_{M}$ as shown in Figure 6b. In the case where the user is later authorized to extract the payload, the user can conduct data extraction from $I_{M}$. In the same way, another user with the privilege to only extract data can extract the payload from the marked encrypted image $I_{ME}$ without decryption and obtain the encrypted image $I_{E}$ as shown in Figure 6c. The content of $I_{E}$ is still concealed by encryption at this stage. Nevertheless, if the user is later authorized to access the image content, the user can decrypt $I_{E}$ and obtain *I*. As pointed out above, the proposed method provides flexible access control corresponding to each user’s request. In contrast, the RDH-MSB method can embed a payload only after encryption and decrypt images only after data extraction. Such inflexible processing can reduce the possibility of practical applications.

Furthermore, we refer to the computational complexity. The RDH-EI methods with high hiding capacity commonly increase the computational complexity compared to the traditional RDH-EI methods with less hiding capacity. Although the proposed method has been extended based on the RDH-BPP method to enhance the hiding capacity, the entire process has been noticeably simplified by introducing a unique algorithm to the self-embedding process. In particular, the prediction values are first calculated by the MED method, and then the embedding process using the prediction error histogram is only iterated. In contrast, the RDH-BPP method has an issue with the self-embedding process. The four modes shown in Figure 2 are used to calculate the prediction values. The RDH-BPP method first predicts the target pixel values under one mode and embeds the original bit values of $I_{2}$ into the prediction values using an efficient algorithm. After finishing a series of processes in the current mode, the same processing is iterated under the next mode. The iterative process is continued until the entire payload is embedded. This algorithm, however, repeatedly uses marked pixels. When using a lot of repetition in this self-embedding process, the prediction accuracy gets worse, which could lead to a vicious circle. This method eventually increases the computational complexity. On another front, the RDH-MSB method embeds prediction error widths to ensure reversibility and does not require any preprocessing including the self-embedding process. This method consists of simple processes without computational complexity.

**Figure 11:**
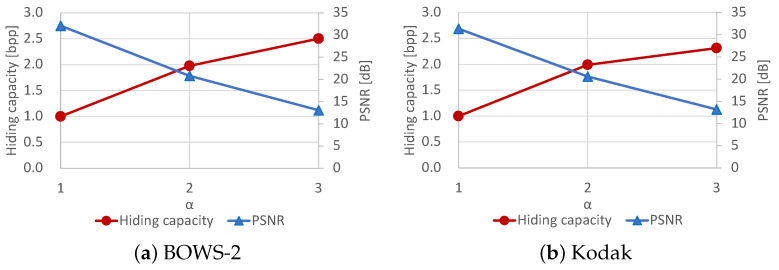
Trade-off between hiding capacity and marked-image quality.

On another front, we also discuss the correlation between the hiding capacity and marked-image quality under different values of α, which specifies the number of bit-planes for data hiding. In general, an increase in the hiding capacity leads to a decrease in the marked-image quality. As mentioned, an original image is divided into $I_{1}$ and $I_{2}$ by bit-plane partition; $I_{1}$ contains α bits of upper bit-planes and is used for encryption, while $I_{2}$ contains 8−α bits of lower bit-planes and is used for data hiding. We can roughly control the hiding capacity with α. However, the minimum capacity is around 1 bpp, and it is difficult for the proposed and RDH-BPP methods to more finely control the capacity. Figure 11 depicts the hiding capacity and PSNR of the marked images when α= 7, 6, or 5, i.e., one, two, or three of the lower bit-planes are used for data hiding. This figure indicates that there exists a trade-off between the hiding capacity and marked-image quality. The proposed method has such a trade-off as with any other method while achieving a high hiding capacity with flexible processing.

Here, we further mention that the hiding capacity depends on α. In the case where one or two bit-planes are adopted for $I_{2}$, almost all of the bits in $I_{2}$ are available for data hiding. However, when three lower bit-planes are used for data hiding, about half of the third lower bit-plane is excluded from the embeddable area. A number of the original bit values of the third bit-plane cannot be embedded into $I_{1}$ in the self-embedding process. This is because, as α gets smaller, $I_{1}$, where the self-embedding process is conducted, it becomes narrow, while $I_{2}$, where the original values are embedded into $I_{1}$, becomes enlarged. Therefore, the embedding efficiency tends to get worse as α decreases.

## 5. Conclusions

We proposed a novel extension of an effective RDH-EI method that independently conducts encryption and data hiding by using area partitions. Our method has two main advantages. One is that it has the highest hiding capacity in this field. The other is that there are no restrictions on the processing order, and thus our method provides flexible processing, which satisfies user request and privilege needs. To this end, we focused on the processing flexibility of a previous RDH-EI method and extended the self-embedding process. This process consists of two processes: prediction and embedding. We introduced the MED method, which has a high prediction accuracy, and applied a refined embedding algorithm based on the original PEE-HS method. Experimental results show that the proposed method achieved the highest hiding capacity and alleviated image distortion in marked images. In addition, the processing flexibility offers image users four restoration options: data extraction only, data extraction then decryption, decryption only, and decryption then data extraction. Furthermore, even when a user is allowed to only decrypt images, the user can also extract the payload later from marked images by obtaining additional privilege. With this feature, the proposed method is expected to be applied to a wide range of applications. As seen in the results, it is clear that the proposed method is one of the best RDH-EI methods ever.

## Figures and Tables

**Table 1:** Average hiding capacity.

	Hiding Capacity [bpp]	
	BOWS-2	Kodak
Proposed	2.50	2.31
RDH-BPP [11]	2.17	1.99
RDH-MSB [14]	2.46	1.78

**Table 2:** Marked-image quality under comparable payload amount.

	Dataset	PSNR [dB]	SSIM	RCE
Proposed	BOWS-2	16.9	0.2060	0.5019 (+0.0019)
	Kodak	17.1	0.3275	0.5227 (+0.0227)
RDH-BPP [11]	BOWS-2	14.0	0.1729	0.5110 (+0.0110)
	Kodak	13.9	0.2322	0.5438 (+0.0438)

**Table 3:** Performance comparison among proposed method and related work [11,14].

	Highest Hiding Capacity	Flexibility of Processing Order	Computational Complexity
Proposed	✓	✓	✓
RDH-BPP [11]	×	✓	×
RDH-MSB [14]	✓ *	×	✓

* Only for BOWS-2 dataset.

## Data Availability

The data presented in this study are available on request from the corresponding author.
